# Dietary intakes of methionine, threonine, lysine, arginine and histidine increased risk of type 2 diabetes in Chinese population: does the mediation effect of obesity exist?

**DOI:** 10.1186/s12889-023-16468-z

**Published:** 2023-08-15

**Authors:** Yuyan Liu, Huan Wang, Yuanhong Liang, Zijun Guo, Litong Qu, Ying Wang, Chengwen Zhang, Guifan Sun, Yongfang Li

**Affiliations:** 1 Department of Clinical Epidemiology, The Fourth Affiliated Hospital of China Medical University, Shenyang, Liaoning China; 2 Key Laboratory of Environmental Stress and Chronic Disease Control & Prevention (China Medical University), Ministry of Education, Shenyang, China; 3 School of Public Health, China Medical University, Shenyang, Liaoning China

**Keywords:** Essential amino acids, Methionine, Threonine, Lysine, Arginine, Histidine, Type 2 diabetes mellitus, Obesity

## Abstract

**Background:**

Published studies have shown positive associations of branched chain and aromatic amino acids with type 2 diabetes mellitus (T2DM), and the findings remain consistent. However, the associations of other essential and semi-essential amino acids, i.e., methionine (Met), threonine (Thr), lysine (Lys), arginine (Arg) and histidine (His), with T2DM remain unknown. Obesity is an important independent risk factor for T2DM, and excessive amino acids can convert into glucose and lipids, which might underlie the associations of amino acids with obesity. Therefore, we aimed to estimate the associations between dietary intakes of these 5 amino acids and T2DM risk, as well as the mediation effects of obesity on these associations, in a Chinese population.

**Methods:**

A total of 10,920 participants (57,293 person-years) were included, and dietary intakes of 5 amino acids were investigated using 24-h dietary recalls. Anthropometric obesity indices were measured at both baseline and the follow-up endpoints. Associations of amino acids with T2DM were estimated using COX regression models, hazard ratios (HRs) and 95% confidence intervals (95% CIs) were shown. The mediation effects of obesity indices were analyzed, and the proportion of the mediation effect was estimated.

**Results:**

Higher intakes of the 5 amino acids were associated with increasing T2DM risk, while significant HRs were only shown in men after adjustments. No interaction by gender was found. Regression analyses using quintiles of amino acids intakes showed that T2DM risk was positively associated with amino acids intakes only when comparing participants with the highest intake levels of amino acids to those with the lowest intake levels. Adjusted correlation coefficients between amino acid intakes and obesity indices measured at follow-up endpoints were significantly positive. Mediation analyses showed that mediation effects of obesity indices existed on associations between amino acids intakes and T2DM risk, and the mediation effect of waist circumference remained strongest for each amino acid.

**Conclusions:**

We found positive associations of dietary intakes of Met, Thr, Lys, Arg and His with increasing T2DM risk in general Chinese residents, on which the mediation effect of obesity existed. These findings could be helpful for developing more constructive guidance in the primary prevention of T2DM based on dietary interventions.

**Supplementary Information:**

The online version contains supplementary material available at 10.1186/s12889-023-16468-z.

## Background

Type 2 diabetes mellitus (T2DM) is an endocrine disorder characterized by high levels of blood glucose and dysfunction of insulin secretion [1]. It has been generally considered that insulin resistance (IR) caused by long-term chronic inflammation might contribute to the occurrence and development of T2DM [2, 3]. Numerous studies have found that the occurrence and development of IR and T2DM could be correlated with the intake of several nutrients, among which amino acids have been reported to have adverse effects on increasing T2DM risk [4–6]. However, the categories of amino acids vary, and for certain amino acids, their associations with T2DM remain controversial.

According to whether de novo synthesis is available or not, amino acids are categorized as essential, semi-essential and nonessential amino acids [7]. Among essential and semi-essential amino acids, for which dietary intake is necessary to obtain an adequate amount of physiological requirements, branched chain (isoleucine, leucine, and valine) and aromatic (phenylalanine and tryptophan) amino acids have shown positive relationships with T2DM in published prospective studies [8–10], and findings remained consistent in different populations, including Chinese [11]. There is a latest meta-analysis revealing that plasma and serum levels of branched chain and aromatic amino acids were positively associated with a higher risk of T2DM [12] and providing concrete evidence on the relationships between these 2 categories of amino acids and T2DM. In contrast, for other essential amino acids, i.e., methionine (Met), threonine (Thr) and lysine (Lys), as well as semi-essential amino acids, i.e., arginine (Arg) and histidine (His), their associations with T2DM remain unknown. For example, Met and Lys were correlated with increasing T2DM risk in a meta-analysis, whereas no association was found for the other 3 amino acids [12]. In a cohort study from the Finnish population, His showed a protective role in decreasing T2DM incidence, while such a relationship was not found in other studies [13, 14]. Simultaneously, controversial results were observed in 2 case–control studies for Arg and Thr [15, 16]. Such inconsistent findings might be attributed to that among these studies, ethnicities, measurements of amino acid levels (blood sample examinations vs. dietary investigations), study designs (case–control vs. cohort), as well as sample sizes varied. Therefore, a prospective study with a large sample size is needed to clarify the relationship between these amino acids and T2DM. In addition, in recent decades, given that the dramatic westernization of dietary patterns has occurred in China and the T2DM incidence continues to increase, the update of diet-related guidelines regarding various nutrients, including amino acids, appears to be of critical importance. Unfortunately, the relationships between intakes of these 5 certain amino acids and T2DM have not been clarified in Chinese populations [17, 18].

On the other hand, relationships between intakes of amino acids and obesity have been revealed previously, while studies referring to intakes of these 5 certain amino acids are limited [8, 19, 20]. It is generally accepted that excessive amino acids from dietary intake can be metabolized into intermediates of the tricarboxylic acid cycle (TCA) [21, 22] and thereafter converted into glucose and lipids [23, 24], which perhaps could underlie the associations of amino acids with obesity. Moreover, obesity is a critical independent risk factor for T2DM, but whether obesity plays a mediating role in the relationships between dietary intakes of amino acids and increasing risk of T2DM has never been disclosed. From an epidemiological perspective, the mediation analysis enables us to explore potential pathways by which exposure factors could be associated with relevant outcomes in population studies. Disclosing the mediation effect of obesity would be helpful to alert people of the increasing risk of T2DM resulted from excessive intakes of amino acids.

Therefore, we performed this longitudinal study aiming to determine the associations of dietary intakes of 5 essential and semi-essential amino acids (i.e., Met, Thr, Lys, Arg and His) with T2DM risk, as well as the mediation effects of obesity on these associations in the general Chinese population.

## Methods

### Study design and populations

**Fig. 1:**
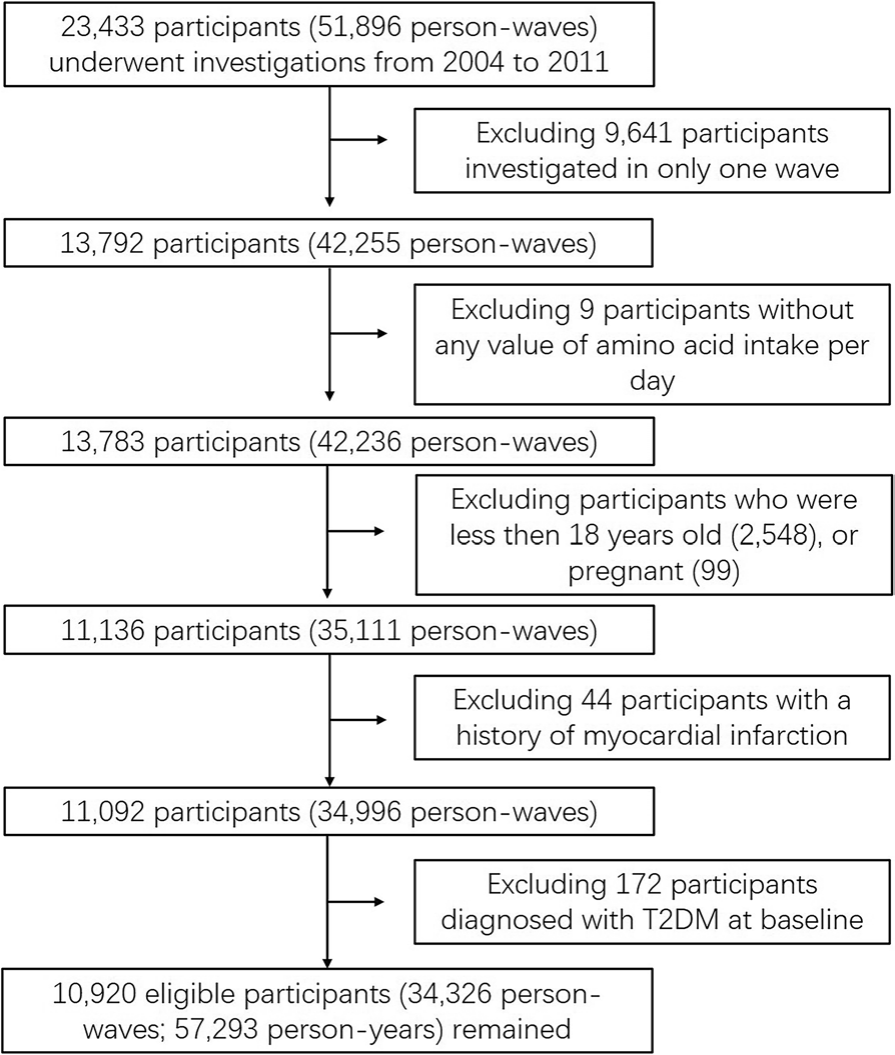
Inclusion and exclusion of participants

The China Health and Nutrition Survey (CHNS) is a large-scale longitudinal study that includes 9 waves of investigations (1989–2011). The main goal of the CHNS is to determine the relationships between health-relevant behaviors and various outcomes among general residents from 15 provinces [25, 26]. In brief, among 23,433 participants (51,896 person-waves) investigated from 2004 to 2011, we selected 13,792 participants (42,255 person-waves) with 2 or more waves (Fig. 1). Thereafter, by following the exclusion criteria, participants younger than 18 years old, pregnant, having a history of myocardial infarction, being diagnosed as T2DM at baseline, and missing data on dietary intakes of amino acids were identified as ineligible. We finally included 10,920 participants (34,326 person-waves, i.e., 57,293 person-years) into the following analyses. The study was conducted in accordance with the Declaration of Helsinki and approved by the Institutional Review Board of the National Institute for Nutrition and Health, Chinese Center for Disease Control and Prevention, China (#201524); and the Carolina Population Center, University of North Carolina, Chapel Hill, NC, USA (#07–1963).

### Information on dietary intake of amino acids

Trained nutritionists collected the dietary information by face-to-face interviews. In this step, a questionnaire investigation of 24-h dietary recalls was performed, and information from the past 3 days was obtained. In the dietary recall investigations, both the categories and amounts of food consumed were recorded. For some specific foods, such as salt and cooking oil, information was collected at the household level. In accordance with the China Food Consumption Tables 2002 and 2004 (FCT 2002 and 2004), 13 food groups were selected to calculate the intake levels of 5 amino acids. The 13 food groups were grains, tubers, soybeans, vegetables, fungi, fruits, seeds and nuts, dairy, red meat, poultry, fish and seafoods, eggs, and oils. For calculating the amount of daily intake of each individual nutrient (including both macronutrients and amino acids), as well as each individual food group, intake values of different time points were summed to obtain the total values, which were then divided by the total number of days consuming these nutrients.

### Definition of T2DM and biochemical examinations

The definition of T2DM was generally based on self-reports, and participants with a history of T2DM diagnosis; and/or receiving any treatment for T2DM (special diet, weight control, oral medication, insulin injection, Chinese traditional medicine, or home remedies) were identified as patients with T2DM. Moreover, after accessing the data of T2DM-related biomarkers, including fasting glucose (FG), insulin and HbA1C, that were available in the wave of 2009, we then defined T2DM as FG ≥ 7.0 mmol/l and/or HbA1c ≥ 6.5% in addition to the diagnostic criteria mentioned above for the specific participants who were investigated in 2009 [27, 28]. Relevant information about the biochemical examinations is briefly summarized as follows. Participants were asked to fast overnight for a blood sample collection (12 ml for each). The glucose oxidase method (Randox, Crumlin, UK) was used to examine serum glucose. Plasma insulin and HbA1c were measured by an ELISA Kit (Millipore Corporation, Billerica, MA, USA) and a whole blood HbA1c HPLC analysis (model HLC-723G7; Tosoh, Tokyo, Japan), respectively.

### Measurements of obesity indices and confounding variables

Anthropometric measurements were performed with participants wearing light clothes and no shoes. A vertical weight scale and a metric scale were used to measure body weight and height and waist circumference (WC), respectively. We calculated body mass index (BMI) and defined overweight or obesity as BMI ≥ 24 kg/m^2^ [29]. WC values of 85 cm and 80 cm were used as cutoff points for central obesity in men and women, respectively [30]. The middle point between the acromion and olecranon processes was selected to measure triceps skinfold thickness (TST). In detail, at the site 2.0 cm above the measuring point, the skin, as well as subcutaneous fat, were grasped, and then the jaws of calipers were placed at the marked level, perpendicular to the skinfold [31, 32]. All of the above 3 measurements of obesity were obtained at both baseline and the follow-up endpoints. Both systolic and diastolic blood pressure (SBP and DBP) were measured using a standard mercury-column sphygmomanometer, and hypertension was diagnosed as SBP/DBP ≥ 140/90 mmHg or the use of antihypertensive drugs [33].

Trained staff collected demographic characteristics using questionnaires, and we included this information as confounders in the analyses, including urban residence, nationality, education level, and status of smoking and alcohol drinking. Among these confounders, we estimated physical activity levels referring to both occupational and home activities and calculated the total metabolic equivalents (METs) of physical activity multiplied by hours per week (MET-hours/week) [34]. Moreover, log-transformation was performed since the distribution of MET-hours/week was strongly skewed in the normal distribution test.

### Statistical analysis

Continuous variables were examined using normal distribution tests, and all of them followed a normal distribution. We then used the mean and standard deviation (SD) to present these variables. We presented categorical variables as percentages. Differences between men and women in continuous and categorical variables were estimated using Student *t*-tests and chi-square tests, respectively. In addition, demographic characteristics were also shown in each of the 5 subgroups, which were divided using quintiles of intakes of the 5 amino acids, and *P*-values for trend were given.

To estimate the relationship between T2DM risk and per SD increase in each amino acid, we performed multivariable COX regression analyses and showed the results using hazard ratio (HR) and 95% confidence interval (95% CI). There were 3 models performed in the regression analysis. Model 1 was adjusted for age; Model 2 was adjusted for nationality, education, urban residents, hypertension, physical activity, smoking and alcohol drinking in addition to Model 1; Model 3 was adjusted for intakes of energy, carbohydrate, fat and protein in addition to Model 2. Based on the quintiles of intakes of 5 amino acids, 5 subgroups were created. Relationships between T2DM risk and categorical variables of dietary intakes of amino acids were then estimated, and subgroups of the lowest intake levels of amino acids were set as the reference. Furthermore, we also performed restricted cubic splines (RCS) analyses aiming to estimate the nonlinearity. For each amino acid, we selected three knots at the 10th, 50th, and 90th centiles and set median values as the reference.

In addition, we performed analyses to determine the interactions by food groups and confounders. All 13 food groups were included, and Spearman correlation analyses were first conducted to estimate correlations between food groups and amino acids. Associations between food groups and T2DM were also estimated in separated and mutual regression models adjusted for confounders. Interactions by several confounding factors were also analyzed, including age, WC, BMI, daily intakes of energy, carbohydrates, fat, and proteins, smoking status, and alcohol drinking.

Moreover, the mediation effects of obesity on the associations of amino acids with T2DM were estimated by running the R package “Mediation”. The mediation analyses were performed using 3 anthropometric obesity indices, i.e., BMI, WC and TST, and all of these measurements were obtained at both baseline and the follow-up endpoints. One point that should be noted was that in the mediation analyses, associations of amino acids with T2DM were estimated using parametric survival analysis based on the accelerated failure time (AFT) model by running the *survreg* function, in which the coefficients were logarithms of ratios of survival time, meaning that a negative coefficient represented shorter survival [35, 36].

In this study, COX regression analyses were run using SAS 9.4 (SAS Institute, Inc., Cary, NC, USA), and mediation analyses were run using R software (v.4.0.3). A *P-*value less than 0.05 was considered to indicate statistical significance.

## Results

### Basic characteristics

The characteristics of the 10,920 participants (5,287 men) are shown in Table 1 as the mean ± SD and percentages. The average ages were 46.9 and 47.7 years old, and the follow-up durations were 5.21 and 5.28 years in men and women, respectively. Although no difference was found in BMI between men and women, men had a significantly larger WC and smaller TST. In our specific participants, we found that intakes of energy, carbohydrate, fat and protein were generally higher in men, as was the intakes of the 5 amino acids. Percentages of smoking and alcohol drinking were dramatically higher in men, and no difference was found for the incidence of T2DM. Characteristics were also shown in the subgroups created using quintiles of intake levels of the 5 amino acids (Table S1).

**Table 1:** Characteristics of men and women (*n* = 10,920)

	Men (*n* = 5,287)	Women (*n* = 5,633)	*P*-value
Age (years old)	46.9 ± 14.8	47.7 ± 14.9	0.003
Follow-up duration (years)	5.21 ± 2.10	5.28 ± 2.12	0.066
Weight (kg)	64.4 ± 10.8	56.2 ± 9.51	< 0.001
Height (cm)	166.8 ± 6.6	155.6 ± 6.4	< 0.001
BMI (kg/m^2^) at baseline	22.9 ± 3.9	23.0 ± 4.0	0.143
WC (cm) at baseline	83.0 ± 9.8	79.5 ± 9.7	< 0.001
TST (mm) at baseline	13.9 ± 8.2	17.4 ± 7.9	< 0.001
BMI (kg/m^2^) at end point of follow-up	23.6 ± 5.0	23.5 ± 4.2	0.200
WC (cm) at end point of follow-up	85.2 ± 10.6	81.8 ± 10.8	< 0.001
TST (mm) at end point of follow-up	14.7 ± 7.5	17.9 ± 6.9	< 0.001
Total physical activity (MET-hours/week)	3.91 ± 1.99	3.19 ± 1.90	< 0.001
Grains (g/day)	706.8 ± 251.8	630.8 ± 238.0	< 0.001
Tubers (g/day)	67.2 ± 91.2	62.8 ± 85.5	0.011
Soybeans (g/day)	91.7 ± 84.5	85.8 ± 80.1	< 0.001
Vegetables (g/day)	579.1 ± 242.1	551.8 ± 225.3	< 0.001
Fungi (g/day)	8.09 ± 18.15	7.88 ± 17.50	0.541
Fruits (g/day)	80.1 ± 130.8	92.1 ± 143.6	< 0.001
Seeds and nuts (g/day)	6.67 ± 18.42	5.98 ± 16.88	0.045
Dairy (g/day)	22.2 ± 61.7	25.2 ± 66.3	0.014
Red meat (g/day)	128.5 ± 93.7	113.3 ± 85.6	< 0.001
Poultry (g/day)	25.9 ± 44.3	23.0 ± 40.4	< 0.001
Fish and seafoods (g/day)	55.6 ± 72.7	50.9 ± 69.1	0.001
Eggs (g/day)	50.8 ± 47.5	49.7 ± 45.8	0.251
Oils (g/day)	38.7 ± 58.9	40.2 ± 54.5	0.185
Energy (Kcal/day)	2370.9 ± 688.0	2011.0 ± 599.9	< 0.001
Carbohydrate (g/day)	342.0 ± 114.2	292.2 ± 100.1	< 0.001
Fat (g/day)	74.5 ± 39.8	65.8 ± 36.3	< 0.001
Protein (g/day)	72.0 ± 25.8	61.8 ± 22.8	< 0.001
Met (g/day)	0.98 ± 0.49	0.90 ± 0.47	< 0.001
Thr (g/day)	2.10 ± 1.01	1.93 ± 0.93	< 0.001
Lys (g/day)	3.79 ± 1.85	3.52 ± 1.76	< 0.001
Arg (g/day)	3.44 ± 1.71	3.15 ± 1.60	< 0.001
His (g/day)	1.50 ± 0.69	1.37 ± 0.62	< 0.001
Urban residents (%)	32.3	36.5	0.826
Han nationality (%)	87.8	87.5	0.791
Education (%)			< 0.001
Illiteracy	9.1	24.2	
Primary school	20.8	22.5	
Middle school	37.6	30.2	
High school or above	32.5	23.1	
Smoking (%)	63.5	4.3	< 0.001
Alcohol drinking (%)	59.7	8.6	< 0.001
Hypertension diagnosed at baseline (%)	7.8	9.1	0.074
Incidence of T2DM (%)	2.9	2.9	0.953

Values of total physical activity were log-transformed

*Abbreviations*: *BMI* body mass index, *WC* waist circumference, *TST* triceps skinfold thickness, *MET* metabolic equivalent, *Met* methionine, *Thr* threonine, *Lys* lysine, *Arg* arginine, *His* histidine, *T2DM* type 2 diabetes mellitus

### Associations of amino acids with T2DM risk

Multivariable COX regression analyses revealed the positive associations of intakes of 5 amino acids with T2DM risk in men after adjusting for age (*P*-values < 0.001), while only Thr, Lys and His were positively associated with T2DM in women, and interactions by gender were found (Table 2). By further adjusting for confounders, the associations of intakes of amino acids with T2DM were attenuated in men but remained statistically significant, while no association was found in women. For example, in Model 3, the HRs for Met, Thr, Lys, Arg and His in men were 1.27 (95% CI: 1.03, 1.58), 1.37 (95% CI: 1.10, 1.71), 1.38 (95% CI: 1.15, 1.65), 1.33 (95% CI: 1.09, 1.62) and 1.48 (95% CI: 1.20, 1.83), respectively. Statistically significant interactions by gender were found only for Lys and His.

**Table 2:** Associations between T2DM incidence and per SD increase of amino acids (*n* = 10,920)

	Men (*n* = 5,287)			Women (*n* = 5,633)			*P*-value for interaction
	HR	95% CI	*P*-value	HR	95% CI	*P*-value	
Model 1							
Met	1.37	1.20, 1.57	< 0.001	1.09	0.99, 1.20	0.082	0.006
Thr	1.42	1.25, 1.63	< 0.001	1.16	1.03, 1.31	0.017	0.025
Lys	1.40	1.25, 1.57	< 0.001	1.12	1.01, 1.24	0.031	0.004
Arg	1.36	1.20, 1.55	< 0.001	1.10	0.99, 1.23	0.064	0.012
His	1.44	1.26, 1.65	< 0.001	1.19	1.04, 1.35	0.010	0.043
Model 2							
Met	1.23	1.02, 1.48	0.030	0.97	0.78, 1.19	0.738	0.069
Thr	1.28	1.07, 1.54	0.007	1.01	0.83, 1.23	0.945	0.050
Lys	1.32	1.12, 1.55	0.001	1.02	0.83, 1.24	0.882	0.037
Arg	1.27	1.07, 1.51	0.006	1.01	0.82, 1.24	0.924	0.058
His	1.35	1.13, 1.61	0.001	1.05	0.86, 1.28	0.625	0.041
Model 3							
Met	1.27	1.03, 1.58	0.026	0.94	0.73, 1.21	0.622	0.068
Thr	1.37	1.10, 1.71	0.005	1.00	0.78, 1.27	0.994	0.050
Lys	1.38	1.15, 1.65	< 0.001	1.01	0.80, 1.28	0.926	0.039
Arg	1.33	1.09, 1.62	0.005	1.00	0.78, 1.30	0.973	0.067
His	1.48	1.20, 1.83	< 0.001	1.06	0.84, 1.34	0.632	0.041

Separate regression models were used for each individual amino acid. Model 1 was adjusted for age; Model 2 was adjusted for nationality, education, urban residents, hypertension, physical activity, smoking and alcohol drinking in addition to Model 1; Model 3 was adjusted for intakes of energy, carbohydrate, fat and protein in addition to Model 2

*Abbreviations*: *T2DM* type 2 diabetes mellitus, *SD* standard deviation, *HR* hazard ratio, *95% CI* 95% confidence interval, *Met* methionine, *Thr* threonine, *Lys* lysine, *Arg* arginine, *His* histidine

**Fig. 2:**
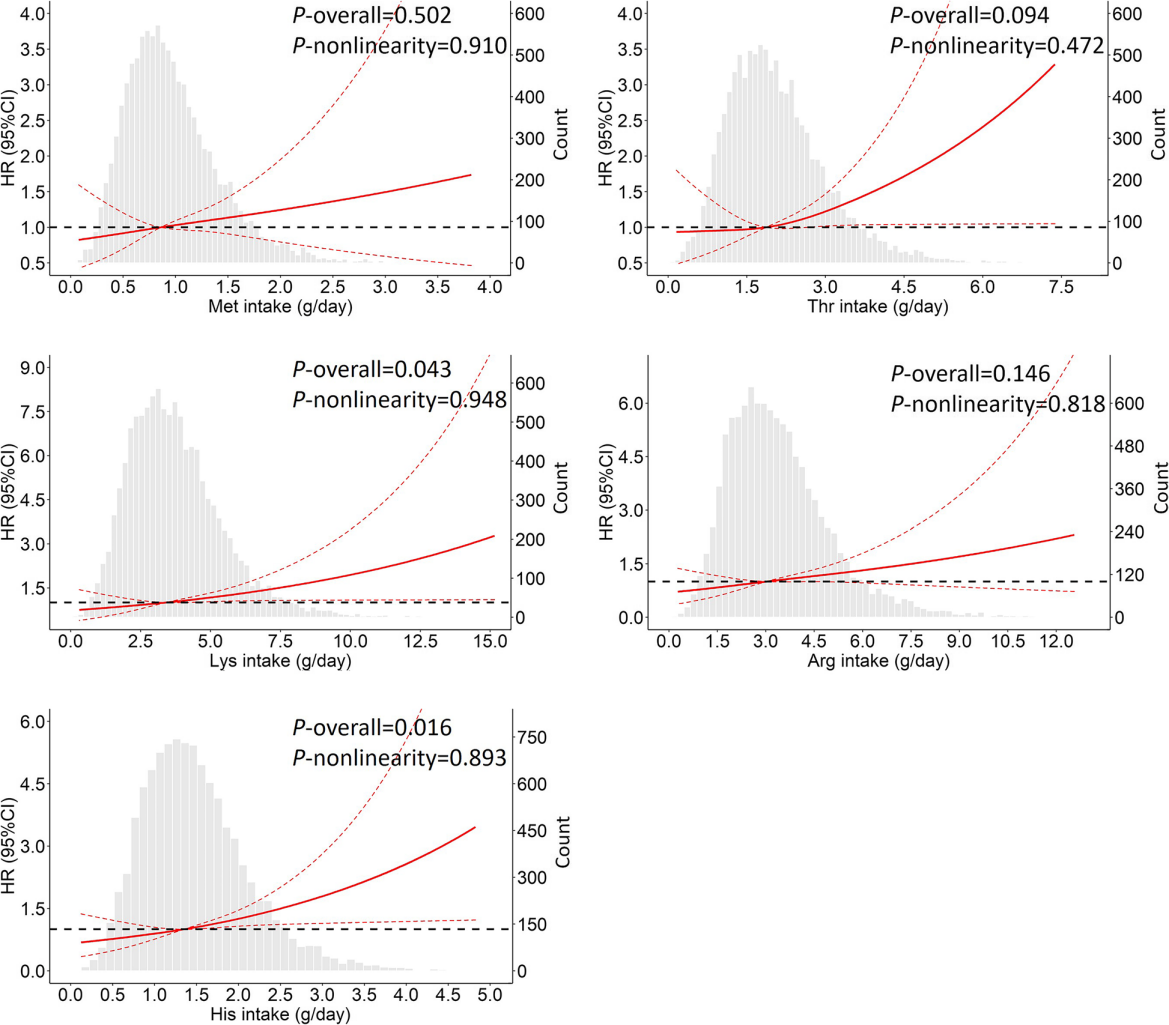
Curves of dose–response relationships between T2DM risk and amino acids. The RCS models were adjusted for age, nationality, education, urban residents, hypertension, physical activity, smoking, alcohol drinking, intakes of energy, carbohydrate, fat and protein. Abbreviations: T2DM: type 2 diabetes mellitus; HR: hazard ratio; 95% CI: 95% confidence interval; Met: methionine; Thr: threonine; Lys: lysine; Arg: arginine; His: histidine

We further divided participants into subgroups according to quintiles of amino acids, and higher T2DM incidence was observed in subgroups with larger intakes of amino acids. However, in men, the HRs in the subgroups of Q5 referred to Q1 were only statistically significant for Lys, Arg, and His (Table 3). As shown in Fig. 2, by performing RCS analyses, no estimate for nonlinearity was statistically significant (*P*-values for nonlinearity > 0.05), suggesting that the relationships between intakes of 5 amino acids and T2DM were linear. As shown in Table S2, we have analyzed associations between dietary amino acid intakes with T2DM risk respectively at different time points, and statistically significant positive associations of dietary intake levels of 5 amino acids with T2DM were only found in 2006, but not in either 2004 or 2009.

**Table 3:** Adjusted associations of T2DM incidence with categorical variables of amino acids based on quintiles (*n* = 10,920)

Men (*n* = 5,287)						Women (*n* = 5,633)						*P*-value for interaction
	n	Cases (incidence)	HR	95% CI	*P*-value		n	Cases (incidence)	HR	95% CI	*P*-value	
Met (g/day)						Met (g/day)						0.281
Q1 (< 0.59)	1,071	23 (2.2%)	Ref			Q1 (< 0.54)	1,143	28 (2.5%)	Ref			
Q2 (0.59–0.80)	1,024	26 (2.5%)	0.90	0.43, 1.89	0.784	Q2 (0.54–0.73)	1,101	26 (2.4%)	0.95	0.49, 1.86	0.886	
Q3 (0.80–1.02)	1,078	25 (2.3%)	0.89	0.43, 1.80	0.736	Q3 (0.73–0.94)	1,148	38 (3.3%)	1.37	0.74, 2.56	0.316	
Q4 (1.02–1.33)	1,047	34 (3.3%)	0.99	0.48, 2.02	0.968	Q4 (0.94–1.22)	1,130	35 (3.1%)	1.21	0.63, 2.31	0.563	
Q5 (≥ 1.33)	1,067	46 (4.3%)	1.46	0.71, 2.99	0.301	Q5 (≥ 1.22)	1,111	36 (3.2%)	0.89	0.42, 1.88	0.758	
Thr (g/day)						Thr (g/day)						0.293
Q1 (< 1.26)	1,053	24 (2.3%)	Ref			Q1 (< 1.17)	1,133	29 (2.6%)	Ref			
Q2 (1.26–1.73)	1,049	20 (1.9%)	0.79	0.37, 1.69	0.547	Q2 (1.17–1.59)	1,105	29 (2.6%)	0.94	0.50, 1.78	0.844	
Q3 (1.73–2.20)	1,068	26 (2.4%)	0.98	0.49, 1.97	0.962	Q3 (1.59–2.02)	1,148	26 (2.3%)	0.96	0.50, 1.84	0.892	
Q4 (2.20–2.82)	1,056	35 (3.3%)	0.82	0.39, 1.71	0.596	Q4 (2.02–2.60)	1,125	41 (3.6%)	1.38	0.75, 2.55	0.297	
Q5 (≥ 2.82)	1,061	49 (4.6%)	1.68	0.83, 3.41	0.147	Q5 (≥ 2.60)	1,122	38 (3.4%)	1.04	0.50, 2.14	0.920	
Lys (g/day)						Lys (g/day)						0.144
Q1 (< 2.28)	1,055	20 (1.9%)	Ref			Q1 (< 2.11)	1,122	27 (2.4%)	Ref			
Q2 (2.28–3.12)	1,063	24 (2.3%)	1.43	0.66, 3.08	0.366	Q2 (2.11–2.89)	1,130	30 (2.7%)	1.06	0.56, 2.01	0.862	
Q3 (3.12–3.95)	1,055	25 (2.4%)	1.43	0.66, 3.07	0.363	Q3 (2.89–3.65)	1,118	26 (2.3%)	1.02	0.52, 1.97	0.964	
Q4 (3.95–5.08)	1,054	34 (3.2%)	1.25	0.56, 2.76	0.586	Q4 (3.65–4.70)	1,134	43 (3.8%)	1.47	0.79, 2.74	0.225	
Q5 (≥ 5.08)	1,060	51 (4.8%)	2.73	1.26, 5.91	0.011	Q5 (≥ 4.70)	1,129	37 (3.3%)	1.06	0.51, 2.21	0.872	
Arg (g/day)						Arg (g/day)						0.150
Q1 (< 2.03)	1,060	20 (1.9%)	Ref			Q1 (< 1.86)	1,134	27 (2.4%)	Ref			
Q2 (2.03–2.80)	1,062	24 (2.3%)	1.78	0.81, 3.88	0.150	Q2 (1.86–2.54)	1,126	29 (2.6%)	1.09	0.58, 2.07	0.787	
Q3 (2.80–3.58)	1,056	26 (2.5%)	1.67	0.76, 3.66	0.198	Q3 (2.54–3.26)	1,113	30 (2.7%)	1.19	0.63, 2.24	0.598	
Q4 (3.58–4.62)	1,052	37 (3.5%)	1.66	0.75, 3.69	0.213	Q4 (3.26–4.21)	1,129	41 (3.6%)	1.34	0.73, 2.55	0.376	
Q5 (≥ 4.62)	1,057	47 (4.5%)	2.96	1.32, 6.61	0.008	Q5 (≥ 4.21)	1,131	36 (3.2%)	1.21	0.58, 2.49	0.615	
His (g/day)						His (g/day)						0.078
Q1 (< 0.94)	1,071	22 (2.1%)	Ref			Q1 (< 0.86)	1,142	29 (2.5%)	Ref			
Q2 (0.94–1.25)	1,028	22 (2.1%)	1.17	0.54, 2.54	0.692	Q2 (0.86–1.15)	1,121	27 (2.4%)	0.82	0.43, 1.57	0.551	
Q3 (1.25–1.58)	1,084	27 (2.5%)	1.19	0.56, 2.54	0.645	Q3 (1.15–1.44)	1,112	29 (2.6%)	0.99	0.53, 1.85	0.962	
Q4 (1.58–1.99)	1,050	33 (3.1%)	1.42	0.67, 3.03	0.366	Q4 (1.44–1.83)	1,132	41 (3.6%)	1.21	0.66, 2.23	0.536	
Q5 (≥ 1.99)	1,054	50 (4.7%)	2.71	1.26, 5.83	0.011	Q5 (≥ 1.83)	1,126	37 (3.3%)	1.12	0.54, 2.30	0.762	

Each individual amino acid was analyzed in separate regression models. Regression models were adjusted for age, nationality, education, urban residents, hypertension, physical activity, smoking, alcohol drinking, intakes of energy, carbohydrate, fat and protein

*Abbreviations*: *T2DM* type 2 diabetes mellitus, *HR* hazard ratio, *95% CI* 95% confidence interval, *Met* methionine, *Thr* threonine, *Lys* lysine, *Arg* arginine, *His* histidine

To determine which food groups contributed to the associations between intakes of 5 amino acids and T2DM, we first performed Spearman correlation analyses between intakes of 5 amino acids and various food groups. As shown in Table S3, the intakes of all food groups except tubers were positively correlated with the 5 amino acids. Tables S4 and S5 showed the results of the associations between these food groups and T2DM risk with and without mutual adjustment respectively. All food groups were first analyzed in separate regression models, and statistically significant positive associations with T2DM risk were found for grains, vegetables, seeds and nuts, red meat, and eggs after adjustments (Table S4). By including all food groups into the same regression model, the intake of seeds and nuts was only found to be positively associated with T2DM risk (Table S5).

Stratified analyses were also conducted among confounders to estimate the interactions. Although no interaction by confounders was found, positive associations of dietary intakes of amino acids with T2DM risk were observed in participants with larger BMI and WC at baseline, higher intakes of carbohydrate, fat, and protein, cigarette smoking and alcohol drinking (Figure S1).

### Mediation effects of obesity on associations of amino acids with T2DM

**Fig. 3:**
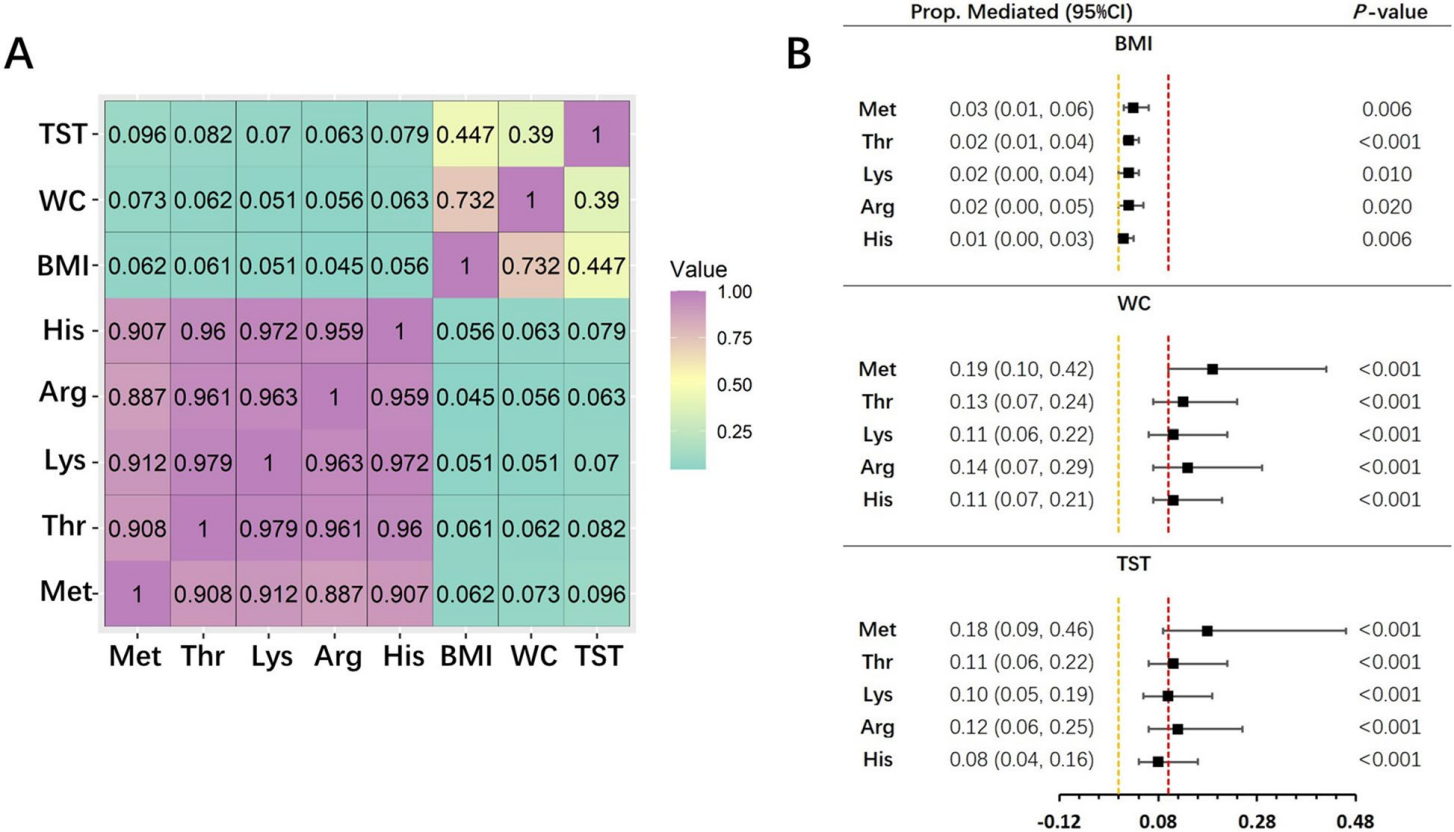
Mediation analyses of anthropometric obesity indices on the associations of amino acids with T2DM. Both Spearman correlation and mediation analyses were adjusted for relevant confounders, including age, gender, physical activity, intakes of energy, carbohydrate, fat and protein; all correlation coefficients shown in **A** were statistically significant (*P*-value < 0.05); in **B**, yellow and red dashed lines respectively represented the points where Prop. Mediated equaled 0.00 and 0.10. Abbreviations: T2DM: type 2 diabetes mellitus; Prop. Mediated: proportion of mediation effect; 95% CI: 95% confidence interval; Met: methionine; Thr: threonine; Lys: lysine; Arg: arginine; His: histidine

Spearman correlation analyses showed that dietary intakes of the 5 amino acids were positively correlated with obesity indices measured at the follow-up endpoints (*P*-values < 0.001, Fig. 3A). The average causal mediation effects (ACME) of 3 obesity indices for all 5 amino acids were negative values with significant *P*-values, implying that mediation effects of obesity existed on the associations of the 5 amino acids with T2DM (Table S6). Compared to BMI, the mediation effects of another 2 obesity indices were generally stronger, especially for WC, whose proportion of mediation effects was generally larger than 0.1 for the 5 amino acids (Fig. 3B). Among the 5 amino acids, the mediation effects of obesity indices were strongest for Met. The proportions of mediation effects of obesity indices on the association between Met and T2DM were 0.03 (95% CI: 0.01, 0.06) for BMI, 0.19 (95% CI: 0.10, 0.42) for WC and 0.18 (95% CI: 0.09, 0.46) for TST. The mediation effects of baseline obesity indices on the association of the average and baseline dietary amino acid intakes and T2DM risk were analyzed (Tables S7 and S8). As shown in Table S7, statistically significant mediation effects of all 3 obesity indices measured at baseline were found on the association between average intake levels of amino acids and T2DM risk. However, no mediation effect was found by using values of baseline dietary intakes of amino acids (Table S8).

## Discussion

In summary, we found positive associations of dietary intakes of Met, Thr, Lys, Arg and His with increasing T2DM risk, especially in men, while no interaction by gender was found. Among the 13 food groups contributing to the dietary intakes of amino acids, the interactions of grain intake on the associations between the 5 amino acids and T2DM were statistically significant. The mediation analyses showed that the mediation effects of obesity on relationships between the 5 amino acids and T2DM existed, and the effects were obvious when WC measurements were used to estimate obesity.

Given that essential amino acids cannot be synthesized in human bodies or that the amount of de novo synthesis is insufficient for biological needs, dietary intake is an important source of essential amino acids to meet physiological requirements [7]. Among essential, as well as semi-essential amino acids, relationships of branched chain and aromatic amino acids with increasing T2DM risk have been frequently reported in numerous population studies, and the conclusions were robust [10]. Even in a Chinese population, dietary intakes of branched chain amino acids were positively associated with increasing T2DM risk [11]. In comparison, for other essential and semi-essential amino acids, i.e., Met, Thr, Lys, Arg and His, population studies on their associations with T2DM are limited, and the results remain inconsistent.

A meta-analysis based on metabolomics data showed that higher plasma and serum levels of Met and Lys were associated with a higher risk of T2DM, while for Thr, Arg and His, no significant result was found [12]. Another cohort study from 4,851 Finnish participants revealed that based on examinations of blood samples, the adjusted positive association with T2DM incidence was only shown for Arg, but not Met, Thr or Lys, and His was inversely associated with T2DM [13]. Azab, et al. performed a case–control study among Canadian children, including 228 individuals with metabolic syndrome (MS) and 228 control individuals, and found that the plasma levels of Met, Thr, Lys and Arg, but not His, were positively associated with MS prevalence [15]. Lee, et al. found that among South Korean participants, the serum levels of Lys, His, and Arg were higher only in those with impaired fasting glucose and T2DM [37]. In a cohort study of 2,139 Iran participants, the intake of L-arginine from dietary investigations was positively associated with T2DM incidence, while the intakes of other amino acids were not estimated [38]. In our prospective cohort study, based on continuous 24-h dietary recall investigations, we found positive associations of dietary intakes of the 5 amino acids with an increased risk of T2DM among 10,920 Chinese participants, which might be the largest sample size among relevant studies to our knowledge. Moreover, before COX regression analyses, we standardized the levels of the 5 amino acids using their SD so that the HRs could be comparable. We found that the adjusted HRs were different for the 5 amino acids, which might be due to the different pathogenesis of each individual amino acid. Such comparative analysis has never been reported in previous studies. We thought that what we found could provide some new directions for future studies of mechanisms exploring which amino acids could have more adverse effects on increasing T2DM risk.

On the other hand, regression analyses using quintiles of dietary intake levels of amino acids showed that T2DM risk was only positively associated with amino acids when comparing participants with the highest levels of amino acids to those with the lowest levels. However, by performing RCS analyses, no nonlinear relationship was shown, i.e., in the ranges of dietary intake levels of amino acids in our specific participants, we did not observe any point from which HRs dramatically increased. Published findings only showed relationships between intakes of various amino acids and T2DM, while the linearity of those relationships has never been analyzed. Our findings of linear relationships based on RCS analyses could provide an accurate understanding in the associations of dietary intakes of 5 amino acids with increasing T2DM risk. Moreover, such findings may partially explain why statistically significant results were only shown in men. As that shown in Table 1, in our specific participants, women showed significantly lower dietary intake levels of amino acids than men, which likely attenuated the relationships in women.

The potential mechanisms underlying the associations of intakes of the 5 amino acids with T2DM remain obscure, and various pathophysiological pathways perhaps exist for each individual amino acid as follows. (1) S-adenosylmethionine, an important metabolite of Met, could perform biological functions as a methyl donor for DNA or protein methylation, which in turn could be connected with glucose metabolism, insulin resistance, and β cell dysfunction. (2) As a glucogenic amino acid, excessive Thr from dietary intake could convert into glucose via glucogenesis. Simultaneously, the ketogenic feature of Thr could make it possible to convert into fatty acids by generating acetyl-CoA, which is an important intermediate of the de novo synthesis of fatty acids [21, 22]. (3) As an important metabolite of Lys, 2-aminoadipic acid (2-AAA) was shown to be closely correlated with IR, which might underlie the positive association between Lys and T2DM incidence [39]. (4) It has been reported that Arg can decrease the cellular uptake of citrulline and then suppress endothelial NO synthase (eNOS) expression and activity, resulting in the development of IR [40, 41]. (5) Koh, et al. published a study in 2018 showing that imidazole propionate produced from His could impair insulin signaling by promoting p62 phosphorylation and thereafter activating mechanistic target of rapamycin complex 1 (mTORC1), which could induce the inhibition of insulin receptor substrate (IRS) phosphorylation and the degradation of IRS [42].

To determine which food groups contributed to the associations between the intakes of amino acids and T2DM, we first performed Spearman correlation analyses between the selected food groups and amino acids. All food groups were significantly correlated with intakes of the 5 amino acids, while inverse correlations were found only for tubers. Based on FCT 2002 and 2004, we noticed that the contents of amino acids in tubers were the lowest among all food groups, and therefore, a high intake of tubers might decrease the consumption of other foods rich in amino acids. We thereafter estimated associations between food groups and T2DM in both separated and mutual regression models, and found that the intake of seeds and nuts was positively associated with T2DM, even including all food groups in the same model. Such findings implied that participants with a higher consumption of seeds and nuts should pay more attention to the increased risk of T2DM due to their higher intakes of amino acids.

Another novel finding in our study was that obesity performed mediation effects on associations of dietary intakes of the 5 amino acids with an increased risk of T2DM. Potential mechanisms underlying such mediation effects could be partially attributed to that glucogenic amino acids, including Met, Thr, Arg and His, can be metabolized into several intermediates of TCA, such as oxaloacetic acid and α-ketoglutaric acid, which are then be converted into glucose via gluconeogenesis [22, 43]. Thereafter, excessive glucose could contribute to the synthesis of fatty acids [44, 45]. On the other hand, ketogenic amino acids, such as Thr and Lys, can be converted into acetyl-CoA, which is the building block of the de novo synthesis of fatty acids [24]. In our specific population, we found that dietary intakes of 5 amino acids were closely correlated with anthropometric obesity indices measured at the follow-up endpoints. Furthermore, statistically significant mediation effects of obesity indices on relationships between amino acids and T2DM were shown. As shown in our findings (Fig. 3), the mediation effects of WC and TST were obviously stronger than those of BMI, implying that the accumulation of fat rather than lean body mass may play a potential role in the associations of dietary intakes of these specific amino acids with increasing T2DM risk. In this section of the mediation analyses, the obesity indices measured at the follow-up endpoints were selected because in our study, the variables of the dietary intakes of amino acids were the average daily intake levels in the overall duration of the follow-up. Therefore, by selecting the measurements of obesity at the follow-up endpoints, the relationship between the long-term dietary intake of amino acids and the obesity risk could be well estimated. Our findings of mediation analyses could provide some evidence for further studies to identify novel potential pathophysiological pathways of amino acids in rising T2DM risk. More importantly, in accordance with what we found, people with excessive intakes of amino acids should maintain sufficient awareness of their body weight, especially regarding central obesity, which might be a signal for an increasing risk of T2DM.

Caution should be taken when interpreting our findings. First, in our study, relevant biochemical indicators of T2DM were only available in the wave of 2009, but not continuously measured, resulting in the effects of dietary intakes of amino acids on those specific biomarkers not being estimated. Second, the lack of data on circulating levels of amino acids limited our ability to validate the accuracy of dietary recall investigations in estimating the intake level of amino acids. Third, considering that this is a study focusing on Chinese residents, for populations with other genetic backgrounds, the generalizability might not be sufficient. Another limitation was that the mediation effects of obesity indices were estimated, and therefore, they could only partially disclose the potential pathogenesis of increasing T2DM risk by an increased intake of amino acids. One strength of our study was that we performed a prospective study with a large sample size, which might be the most representative in the field of exploring the relationship between amino acids and T2DM. Second, we calculated the intake levels of amino acids at different time points and then averaged them. We thought the effect of continuous dietary intakes could be well reflected using the averaged values.

## Conclusions

We found linear associations of dietary intakes of Met, Thr, Lys, Arg, and His with increasing T2DM risk in general Chinese residents, on which the mediation effect of obesity existed. What we found implied that dietary intakes of these 5 amino acids may increase T2DM risk by resulting in the accumulation of fat. Such findings would be helpful in developing some favorable dietary guidance in controlling T2DM risk.

### Supplementary Information

**Additional file 1: Table S1.** General characteristics of participants (*n*=10,920). **Table S2.** Adjusted associations of T2DM incidence with intake levels of amino acids in different time points. **Table S3.** Spearman correlation analyses between amino acids and food groups (*n*=10,920). **Table S4.** Adjusted associations of T2DM incidence with intake levels of food groups (*n*=10,920). **Table S5.** Adjusted associations of T2DM incidence with intake levels of food groups in the mutual model (*n*=10,920). **Table S6.** Mediation effects of obesity estimated by anthropometric measurements on the association between amino acids and T2DM. **Table S7.** Mediation effects of obesity estimated by anthropometric measurements at baseline on the association between average intake levels of amino acids and T2DM. **Table S8.** Mediation effects of obesity estimated by anthropometric measurements at baseline on the association between intake levels of amino acids collected at baseline and T2DM. **Figure S1.** Stratified analyses of relationship between dietary intakes of amino acids and T2DM based on confounders. Regression models were adjusted for age, nationality, education, urban residents, hypertension, physical activity, smoking, alcohol drinking, intakes of energy, carbohydrate, fat and protein.

## Data Availability

The datasets analyzed for this study can be found in China Health and Nutrition Survey (https://www.cpc.unc.edu/projects/china).

## Abbreviations

BMI: Body mass index
WC: Waist circumference
TST: Triceps skinfold thickness
MET: Metabolic equivalent
Met: Methionine
Thr: Threonine
Lys: Lysine
Arg: Arginine
His: Histidine
T2DM: Type 2 diabetes mellitus
HR: Hazard ratio
95% CI: 95% Confidence interval
Prop. Mediated: Proportion of mediation effect
