# Supplementary material for: Dietary intakes of methionine, threonine, lysine, arginine and histidine increased risk of type 2 diabetes in Chinese population: does the mediation effect of obesity exist?
Source: BMC Public Health. 2023 Aug 15;23:1551. doi: 10.1186/s12889-023-16468-z (PMC10428589; doi:10.1186/s12889-023-16468-z)
Supplement: Supplementary file 1 — Additional file 1: Table S1. General characteristics of participants (n=10,920). Table S2. Adjusted associations of T2DM incidence with intake levels of amino acids in different time points. Table S3. Spearman correlation analyses between amino acids and food groups (n=10,920). Table S4. Adjusted associations of T2DM incidence with intake levels of food groups (n=10,920). Table S5. Adjusted associations of T2DM incidence with intake levels of food groups in the mutual model (n=10,920). Table S6. Mediation effects of obesity estimated by anthropometric measurements on the association between amino acids and T2DM. Table S7. Mediation effects of obesity estimated by anthropometric measurements at baseline on the association between average intake levels of amino acids and T2DM. Table S8. Mediation effects of obesity estimated by anthropometric measurements at baseline on the association between intake levels of amino acids collected at baseline and T2DM. Figure S1. Stratified analyses of relationship between dietary intakes of amino acids and T2DM based on confounders. Regression models were adjusted for age, nationality, education, urban residents, hypertension, physical activity, smoking, alcohol drinking, intakes of energy, carbohydrate, fat and protein. [file 12889_2023_16468_MOESM1_ESM.docx]

**Dietary intakes of methionine, threonine, lysine, arginine and histidine increased risk of type 2 diabetes in Chinese population: does the mediation effect of obesity exist?**

**No. of tables:** 8 tables and 1 figure

| **Table S1.** General characteristics of participants (n=10,920) | | | | | | |
| --- | --- | --- | --- | --- | --- | --- |
|  | Intake level of 5 amino acids per day | | | | | *P*-value for trend |
|  | Q1 (<6.8 g/day) | Q2 (6.8-9.3 g/day) | Q3 (9.3-11.8 g/day) | Q4 (11.8-15.2 g/day) | Q5 (>15.2 g/day) |  |
|  | (n=2,184) | (n=2,187) | (n=2,177) | (n=2,186) | (n=2,186) |  |
| Age (years old) | 48.7±16.2 | 46.9±14.9 | 47.1±14.7 | 46.7±14.5 | 47.0±13.8 | <0.001 |
| Follow-up duration (years) | 5.14±2.18 | 5.26±2.14 | 5.44±2.06 | 5.31±2.04 | 5.08±2.11 | 0.607 |
| Weight (kg) | 57.7±11.0 | 59.0±10.6 | 59.8±10.7 | 61.4±10.8 | 62.5±11.1 | <0.001 |
| Height (cm) | 159.1±8.8 | 160.2±8.5 | 160.7±8.3 | 161.9±8.3 | 162.8±8.3 | <0.001 |
| BMI (kg/m^2^) at baseline | 22.5±4.1 | 22.7±4.0 | 23.0±3.8 | 23.2±3.9 | 23.3±3.9 | <0.001 |
| WC (cm) at baseline | 80.0±10.2 | 80.3±9.8 | 81.0±9.8 | 81.8±9.8 | 82.7±9.7 | <0.001 |
| TST (mm) at baseline | 15.1±8.7 | 15.2±8.4 | 16.0±8.2 | 16.2±7.7 | 16.3±8.0 | <0.001 |
| BMI (kg/m^2^) at end point of follow-up | 23.1±4.7 | 23.5±5.6 | 23.5±4.3 | 23.8±4.1 | 24.0±4.2 | <0.001 |
| WC (cm) at end point of follow-up | 82.3±10.9 | 82.5±11.1 | 83.3±10.5 | 84.0±10.9 | 85.0±10.4 | <0.001 |
| TST (mm) at end point of follow-up | 15.1±7.1 | 16.2±7.3 | 16.3±7.1 | 16.8±7.8 | 17.5±7.3 | <0.001 |
| Total physical activity (MET-hours/week) | 3.48±2.07 | 3.51±2.01 | 3.47±1.95 | 3.46±1.93 | 3.67±1.89 | 0.016 |
| Grains (g/day) | 620.5±225.0 | 645.9±230.3 | 647.2±209.8 | 653.9±218.0 | 766.5±312.3 | <0.001 |
| Tubers (g/day) | 78.9±102.2 | 67.8±89.2 | 65.9±94.1 | 57.9±78.7 | 55.1±73.1 | <0.001 |
| Soybeans (g/day) | 59.7±62.3 | 70.9±73.6 | 89.3±74.8 | 96.6±81.9 | 124.6±97.6 | <0.001 |
| Vegetables (g/day) | 507.3±223.9 | 524.4±215.0 | 551.5±214.7 | 584.6±218.3 | 652.7±264.5 | <0.001 |
| Fungi (g/day) | 3.8±11.4 | 5.9±14.8 | 7.8±16.3 | 10.2±20.6 | 12.0±22.2 | <0.001 |
| Fruits (g/day) | 64.0±106.9 | 74.0±112.0 | 88.1±131.3 | 100.0±165.4 | 104.0±156.8 | <0.001 |
| Seeds and nuts (g/day) | 2.1±7.9 | 3.8±12.0 | 5.8±14.9 | 7.6±16.6 | 12.0±27.9 | <0.001 |
| Dairy (g/day) | 7.5±31.6 | 15.8±49.8 | 23.4±61.5 | 32.8±76.2 | 38.2±81.9 | <0.001 |
| Red meat (g/day) | 60.5±53.6 | 95.6±65.2 | 122.2±72.6 | 150.5±86.6 | 170.4±112.7 | <0.001 |
| Poultry (g/day) | 9.6±22.5 | 16.1±28.2 | 23.4±37.0 | 30.3±42.4 | 41.8±61.6 | <0.001 |
| Fish and seafoods (g/day) | 16.9±27.6 | 33.0±42.5 | 47.7±53.3 | 67.9±67.0 | 97.9±104.3 | <0.001 |
| Eggs (g/day) | 39.9±46.0 | 44.5±38.0 | 46.9±39.5 | 56.0±46.2 | 63.0±57.0 | <0.001 |
| Oils (g/day) | 34.2±57.5 | 38.9±92.0 | 37.5±27.7 | 42.0±42.0 | 44.7±42.3 | <0.001 |
| Energy (Kcal/day) | 1871.2±609.0 | 2019.8±578.7 | 2148.5±575.5 | 2280.2±626.4 | 2585.8±711.8 | <0.001 |
| Carbohydrate (g/day) | 296.7±107.4 | 305.4±101.6 | 309.8±100.5 | 314.0±105.3 | 354.2±124.1 | <0.001 |
| Fat (g/day) | 51.9±34.9 | 60.9±31.9 | 70.2±32.9 | 78.6±37.7 | 87.3±42.3 | <0.001 |
| Protein (g/day) | 50.2±16.1 | 58.1±17.2 | 64.4±18.0 | 72.1±20.6 | 87.8±30.6 | <0.001 |
| Met (g/day) | 0.43±0.13 | 0.68±0.11 | 0.87±0.13 | 1.11±0.17 | 1.62±0.49 | <0.001 |
| Thr (g/day) | 0.90±0.26 | 1.44±0.17 | 1.88±0.17 | 2.38±0.20 | 3.49±0.83 | <0.001 |
| Lys (g/day) | 1.63±0.44 | 2.61±0.27 | 3.40±0.28 | 4.31±0.36 | 6.31±1.76 | <0.001 |
| Arg (g/day) | 1.45±0.38 | 2.31±0.25 | 3.04±0.30 | 3.87±0.39 | 5.77±1.57 | <0.001 |
| His (g/day) | 0.67±0.18 | 1.05±0.12 | 1.35±0.12 | 1.69±0.16 | 2.42±0.55 | <0.001 |
| Urban residents (%) | 25.8 | 32.4 | 40.3 | 44.6 | 39.1 | <0.001 |
| Han nationality (%) | 79.5 | 84.8 | 87.3 | 91.3 | 95.2 | <0.001 |
| Education (%) |  |  |  |  |  | <0.001 |
| Illiteracy | 21 | 14.7 | 15.9 | 14.9 | 18.3 |  |
| Primary school | 28.2 | 21.8 | 21.9 | 17.2 | 19.5 |  |
| Middle school | 31.8 | 36.2 | 33.5 | 34.5 | 32.7 |  |
| High school or above | 19 | 27.3 | 28.7 | 33.4 | 29.5 |  |
| Smoking (%) | 30.6 | 30.4 | 34 | 31.7 | 37.6 | <0.001 |
| Alcohol drinking (%) | 27.3 | 29.2 | 34.3 | 35.7 | 40.2 | <0.001 |
| Hypertension diagnosed at baseline (%) | 8.9 | 7.8 | 7.2 | 8.1 | 10.3 | 0.516 |
| Incidence of T2DM (%) | 2.2 | 2.5 | 2.3 | 3.6 | 3.9 | <0.001 |
| Values are presented as mean ± SD, or %; values of total physical activity were log-transformed. Abbreviations: BMI: body mass index; WC: waist circumference; TST: triceps skinfold thickness; MET: metabolic equivalent; Met: methionine; Thr: threonine; Lys: lysine; Arg: arginine; His: histidine; T2DM: type 2 diabetes mellitus. | | | | | | |

| **Table S2.** Adjusted associations of T2DM incidence with intake levels of amino acids in different time points | | | |
| --- | --- | --- | --- |
|  | HR | 95% CI | *P*-value |
| 2004 (n=8,402) | | | |
| Met | 0.95 | 0.81, 1.01 | 0.480 |
| Thr | 0.99 | 0.92, 1.06 | 0.691 |
| Lys | 1.00 | 0.96, 1.03 | 0.830 |
| Arg | 0.99 | 0.96, 1.03 | 0.737 |
| His | 1.02 | 0.91, 1.12 | 0.830 |
| 2006 (n=8,933) | | | |
| Met | 1.33 | 1.07, 1.64 | 0.009 |
| Thr | 1.15 | 1.04, 1.27 | 0.007 |
| Lys | 1.08 | 1.03, 1.15 | 0.004 |
| Arg | 1.08 | 1.02, 1.15 | 0.013 |
| His | 1.26 | 1.09, 1.46 | 0.002 |
| 2009 (n=9.140) | | | |
| Met | 0.91 | 0.65, 1.27 | 0.574 |
| Thr | 0.98 | 0.84, 1.15 | 0.818 |
| Lys | 0.99 | 0.91, 1.07 | 0.722 |
| Arg | 0.99 | 0.91, 1.08 | 0.833 |
| His | 0.96 | 0.76, 1.21 | 0.701 |
| Each individual amino acid was analyzed in separate regression models. Models were adjusted for age, nationality, education, urban residents, hypertension, physical activity, smoking, alcohol drinking, energy, carbohydrate, fat and protein. Abbreviations: T2DM: type 2 diabetes mellitus; SD: standard deviation; HR: hazard ratio; 95% CI: 95% confidence interval; Met: methionine; Thr: threonine; Lys: lysine; Arg: arginine; His: histidine. | | | |

| **Table S3.** Spearman correlation analyses between amino acids and food groups (n=10,920) | | | | | |
| --- | --- | --- | --- | --- | --- |
|  | Met | Thr | Lys | Arg | His |
| Grains | 0.172 | 0.134 | 0.146 | 0.16 | 0.19 |
| Tubers | -0.058 | -0.053 | -0.063 | -0.058 | -0.048 |
| Soybeans | 0.214 | 0.288 | 0.298 | 0.319 | 0.296 |
| Vegetables | 0.208 | 0.22 | 0.236 | 0.237 | 0.229 |
| Fungi | 0.18 | 0.187 | 0.178 | 0.174 | 0.163 |
| Fruits | 0.109 | 0.114 | 0.099 | 0.077 | 0.068 |
| Seeds and nuts | 0.198 | 0.21 | 0.202 | 0.262 | 0.196 |
| Dairy | 0.227 | 0.217 | 0.219 | 0.18 | 0.186 |
| Red meat | 0.37 | 0.432 | 0.458 | 0.406 | 0.447 |
| Poultry | 0.23 | 0.248 | 0.25 | 0.224 | 0.22 |
| Fish and seafoods | 0.421 | 0.397 | 0.398 | 0.362 | 0.312 |
| Eggs | 0.241 | 0.206 | 0.194 | 0.169 | 0.176 |
| Oils | 0.216 | 0.214 | 0.232 | 0.235 | 0.226 |
| All correlation coefficients were statistically significant (*P*-value < 0.05). **Abbreviations:** Met: methionine; Thr: threonine; Lys: lysine; Arg: arginine; His: histidine. | | | | | |

| **Table S4.** Adjusted associations of T2DM incidence with intake levels of food groups (n=10,920) | | | |
| --- | --- | --- | --- |
|  | HR | 95% CI | *P*-value |
| Grains | 1.64 | 1.05, 2.57 | 0.029 |
| Tubers | 1.14 | 0.97, 1.34 | 0.119 |
| Soybeans | 1.17 | 0.98, 1.38 | 0.076 |
| Vegetables | 1.94 | 1.36, 2.76 | <0.001 |
| Fungi | 1.22 | 0.97, 1.53 | 0.090 |
| Fruits | 0.94 | 0.81, 1.10 | 0.456 |
| Seeds and nuts | 1.34 | 1.03, 1.73 | 0.027 |
| Dairy | 1.15 | 0.94, 1.40 | 0.182 |
| Red meat | 1.36 | 1.12, 1.65 | 0.002 |
| Poultry | 1.13 | 0.89, 1.44 | 0.330 |
| Fish and seafoods | 1.08 | 0.92, 1.27 | 0.354 |
| Eggs | 1.37 | 1.12, 1.69 | 0.003 |
| Oils | 1.00 | 0.77, 1.31 | 0.999 |
| All food groups were analyzed in separate regression models. Models were adjusted for age, nationality, education, urban residents, hypertension, physical activity, smoking, alcohol drinking, energy, carbohydrate, fat and protein. Abbreviations: T2DM: type 2 diabetes mellitus; HR: hazard ratio; 95% CI: 95% confidence interval. | | | |

| **Table S5.** Adjusted associations of T2DM incidence with intake levels of food groups in the mutual model (n=10,920) | | | |
| --- | --- | --- | --- |
|  | HR | 95% CI | *P*-value |
| Grains | 4.46 | 0.09, 231.49 | 0.458 |
| Tubers | 0.64 | 0.24, 1.74 | 0.380 |
| Soybeans | 0.66 | 0.13, 3.34 | 0.619 |
| Vegetables | 0.33 | 0.01, 15.81 | 0.576 |
| Fungi | 3.11 | 0.83, 11.69 | 0.093 |
| Fruits | 0.47 | 0.17, 1.30 | 0.147 |
| Seeds and nuts | 7.74 | 1.30, 46.19 | 0.025 |
| Dairy | 1.33 | 0.35, 5.10 | 0.678 |
| Red meat | 0.42 | 0.05, 3.35 | 0.410 |
| Poultry | 0.64 | 0.18, 2.30 | 0.490 |
| Fish and seafoods | 0.66 | 0.24, 1.82 | 0.423 |
| Eggs | 1.16 | 0.24, 5.58 | 0.857 |
| Oils | 0.26 | 0.02, 3.76 | 0.320 |
| All food groups were included in the same regression models. Models were adjusted for age, nationality, education, urban residents, hypertension, physical activity, smoking, alcohol drinking, energy, carbohydrate, fat and protein. Abbreviations: T2DM: type 2 diabetes mellitus; SD: standard deviation; HR: hazard ratio; 95% CI: 95% confidence interval. | | | |

| **Table S6.** Mediation effects of obesity estimated by anthropometric measurements on the association between amino acids and T2DM | | | | | |
| --- | --- | --- | --- | --- | --- |
|  | ACME (95%CI) | ADE (95%CI) | Total effect (95%CI) | Prop. Mediated (95%CI) | *P*-value |
|  | BMI | | | | |
| Met | -0.11 (-0.24, -0.03) | -4.62 (-7.94, -1.55) | -4.74 (-8.06, -1.59) | 0.03 (0.01, 0.06) | 0.006 |
| Thr | -0.09 (-0.16, -0.04) | -4.49 (-7.33, -2.17) | -4.58 (-7.32, -2.17) | 0.02 (0.01, 0.04) | <0.001 |
| Lys | -0.03 (-0.07, -0.01) | -1.91 (-3.21, -0.87) | -1.94 (-3.23, -0.89) | 0.02 (0.00, 0.04) | 0.010 |
| Arg | -0.03 (-0.06, -0.01) | -1.69 (-2.98, -0.57) | -1.72 (-3.00, -0.57) | 0.02 (0.00, 0.05) | 0.020 |
| His | -0.11 (-0.22, -0.03) | -8.65 (-14.52, -4.05) | -8.77 (-14.66, -4.14) | 0.01 (0.00, 0.03) | 0.006 |
|  | WC | | | | |
| Met | -1.38 (-2.25, -0.75) | -5.79 (-10.48, 1.84) | -7.18 (-12.26, -3.19) | 0.19 (0.10, 0.42) | <0.001 |
| Thr | -0.78 (-1.28, -0.40) | -5.27 (-9.21, -2.22) | -6.04 (-10.36, -2.83) | 0.13 (0.07, 0.24) | <0.001 |
| Lys | -0.31 (-0.52, -0.14) | -2.37 (-3.93, -1.08) | -2.68 (-4.38, -1.31) | 0.11 (0.06, 0.22) | <0.001 |
| Arg | -0.35 (-0.60, -0.16) | -2.11 (-3.75, -0.73) | -2.46 (-4.17, -1.01) | 0.14 (0.07, 0.29) | <0.001 |
| His | -1.2 (-1.92, -0.65) | -9.6 (-16.76, -4.32) | -10.8 (-18.41, -5.17) | 0.11 (0.07, 0.21) | <0.001 |
|  | TST | | | | |
| Met | -1.04 (-1.65, -0.54) | -4.68 (-8.71, -1.08) | -5.71 (-9.86, -1.83) | 0.18 (0.09, 0.46) | <0.001 |
| Thr | -0.55 (-0.87, -0.29) | -4.49 (-7.58, -1.80) | -5.04 (-8.29, -2.22) | 0.11 (0.06, 0.22) | <0.001 |
| Lys | -0.23 (-0.38, -0.11) | -2.01 (-3.38, -0.94) | -2.24 (-3.65, -1.08) | 0.10 (0.05, 0.19) | <0.001 |
| Arg | -0.23 (-0.39, -0.11) | -1.78 (-3.22, -0.61) | -2.01 (-3.45, -0.78) | 0.12 (0.06, 0.25) | <0.001 |
| His | -0.76 (-1.26, -0.37) | -8.52 (-14.44, -3.86) | -9.28 (-15.62, -4.49) | 0.08 (0.04, 0.16) | <0.001 |
| Regression models were adjusted for age, gender, physical activity, intakes of energy, carbohydrate, fat and protein. Abbreviations: T2DM: type 2 diabetes mellitus; ACME: average causal mediation effect; ADE: average direct effect; Prop. Mediated: proportion of mediation effect; 95% CI: 95% confidence interval; Met: methionine; Thr: threonine; Lys: lysine; Arg: arginine; His: histidine. | | | | | |

| **Table S7.** Mediation effects of obesity estimated by anthropometric measurements at baseline on the association between average intake levels of amino acids and T2DM | | | | | |
| --- | --- | --- | --- | --- | --- |
|  | ACME (95%CI) | ADE (95%CI) | Total effect (95%CI) | Prop. Mediated (95%CI) | *P*-value |
| BMI | | | | | |
| Met | -1.46 (-2.52, -0.64) | -5.27 (-10.69, -0.40) | -6.73 (-12.79, -1.50) | 0.22 (0.09, 0.73) | 0.004 |
| Thr | -0.92 (-1.62, -0.41) | -5.33 (-10.14, -1.81) | -6.26 (-11.42, -2.58) | 0.15 (0.08, 0.32) | <0.001 |
| Lys | -0.37 (-0.70, -0.13) | -2.70 (-4.95, -1.08) | -3.07 (-5.52, -1.33) | 0.12 (0.05, 0.26) | 0.004 |
| Arg | -0.34 (-0.69, -0.09) | -2.10 (-3.89, -0.62) | -2.44 (-4.36, -0.96) | 0.14 (0.04, 0.36) | 0.008 |
| His | -1.15 (-2.07, -0.41) | -9.98 (-17.44, -4.33) | -11.13 (-19.06, -5.11) | 0.10 (0.05, 0.21) | <0.001 |
| WC | | | | | |
| Met | -2.44 (-3.84, -1.41) | -5.36 (-11.54, -0.44) | -7.80 (-14.52, -2.64) | 0.31 (0.17, 0.81) | 0.004 |
| Thr | -1.32 (-2.13, -0.74) | -5.51 (-10.16, -1.96) | -6.83 (-11.91, -2.84) | 0.19 (0.11, 0.37) | <0.001 |
| Lys | -0.54 (-0.89, -0.24) | -2.87 (-5.23, -1.04) | -3.41 (-6.05, -1.48) | 0.16 (0.08, 0.31) | <0.001 |
| Arg | -0.55 (-0.94, -0.26) | -2.22 (-4.39, -0.57) | -2.76 (-5.15, -1.02) | 0.20 (0.10, 0.46) | <0.001 |
| His | -1.81 (-3.05, -0.91) | -9.74 (-17.80, -3.77) | -11.55 (-20.50, -5.06) | 0.16 (0.09, 0.29) | <0.001 |
| TST | | | | | |
| Met | -0.63 (-1.09, -0.28) | -5.00 (-9.37, -1.02) | -5.63 (-10.15, -1.57) | 0.11 (0.05, 0.32) | 0.006 |
| Thr | -0.43 (-0.73, -0.20) | -5.06 (-8.86, -2.18) | -5.49 (-9.44, -2.49) | 0.08 (0.04, 0.15) | <0.001 |
| Lys | -0.19 (-0.33, -0.08) | -2.20 (-3.76, -1.02) | -2.38 (-4.03, -1.15) | 0.08 (0.04, 0.15) | <0.001 |
| Arg | -0.20 (-0.35, -0.09) | -1.96 (-3.49, -0.66) | -2.16 (-3.72, -0.85) | 0.09 (0.04, 0.22) | <0.001 |
| His | -0.43 (-0.86, -0.15) | -9.40 (-15.59, -4.56) | -9.83 (-16.30, -4.90) | 0.04 (0.02, 0.09) | <0.001 |
| Regression models were adjusted for age, gender, physical activity, intakes of energy, carbohydrate, fat and protein. Abbreviations: T2DM: type 2 diabetes mellitus; ACME: average causal mediation effect; ADE: average direct effect; Prop. Mediated: proportion of mediation effect; 95% CI: 95% confidence interval; Met: methionine; Thr: threonine; Lys: lysine; Arg: arginine; His: histidine. | | | | | |

| **Table S8.** Mediation effects of obesity estimated by anthropometric measurements at baseline on the association between intake levels of amino acids collected at baseline and T2DM | | | | | |
| --- | --- | --- | --- | --- | --- |
|  | ACME (95%CI) | ADE (95%CI) | Total effect (95%CI) | Prop. Mediated (95%CI) | *P*-value |
| BMI | | | | | |
| Met | -0.12 (-0.55, 0.25) | 1.85 (-1.76, 5.40) | 1.73 (-1.88, 5.13) | -0.04 (-0.74, 1.12) | 0.700 |
| Thr | -0.16 (-0.38, 0.04) | 0.53 (-1.19, 2.10) | 0.37 (-1.40, 2.01) | -0.11 (-2.50, 2.32) | 0.660 |
| Lys | -0.04 (-0.15, 0.05) | 0.20 (-0.70, 1.00) | 0.15 (-0.72, 1.00) | -0.03 (-2.36, 1.63) | 0.890 |
| Arg | -0.02 (-0.13, 0.09) | 0.23 (-0.65, 1.13) | 0.21 (-0.68, 1.11) | -0.01 (-1.33, 1.39) | 0.920 |
| His | -0.21 (-0.55, 0.10) | 0.02 (-2.61, 2.61) | -0.19 (-2.93, 2.44) | 0.04 (-2.62, 3.16) | 0.910 |
| WC | | | | | |
| Met | -0.67 (-1.17, -0.27) | 2.16 (-1.86, 5.54) | 1.48 (-2.54, 4.94) | -0.25 (-3.75, 3.24) | 0.402 |
| Thr | -0.48 (-0.79, -0.25) | 0.57 (-1.26, 2.42) | 0.09 (-1.75, 1.85) | -0.26 (-6.13, 11.55) | 0.920 |
| Lys | -0.18 (-0.34, -0.07) | 0.23 (-0.75, 1.15) | 0.05 (-0.94, 0.97) | -0.16 (-6.09, 7.13) | 0.910 |
| Arg | -0.18 (-0.34, -0.06) | 0.27 (-0.77, 1.16) | 0.09 (-0.97, 0.97) | -0.19 (-6.62, 4.99) | 0.830 |
| His | -0.75 (-1.27, -0.39) | 0.15 (-2.45, 2.44) | -0.60 (-3.37, 1.82) | 0.40 (-10.35, 8.67) | 0.680 |
| TST | | | | | |
| Met | -0.06 (-0.22, 0.08) | 0.63 (-2.29, 3.49) | 0.57 (-2.31, 3.46) | -0.01 (-0.73, 0.80) | 0.840 |
| Thr | -0.08 (-0.17, 0.00) | 0.09 (-1.42, 1.41) | 0.01 (-1.46, 1.32) | -0.02 (-1.57, 2.56) | 0.938 |
| Lys | -0.02 (-0.06, 0.02) | 0.02 (-0.77, 0.70) | 0.00 (-0.78, 0.69) | 0.00 (-0.96, 1.33) | 0.990 |
| Arg | -0.03 (-0.08, 0.01) | 0.09 (-0.71, 0.82) | 0.06 (-0.75, 0.79) | -0.02 (-1.25, 1.09) | 0.910 |
| His | -0.09 (-0.23, 0.03) | -0.56 (-2.86, 1.53) | -0.65 (-2.95, 1.46) | 0.05 (-1.30, 0.98) | 0.650 |
| Regression models were adjusted for age, gender, physical activity, intakes of energy, carbohydrate, fat and protein. Abbreviations: T2DM: type 2 diabetes mellitus; ACME: average causal mediation effect; ADE: average direct effect; Prop. Mediated: proportion of mediation effect; 95% CI: 95% confidence interval; Met: methionine; Thr: threonine; Lys: lysine; Arg: arginine; His: histidine. | | | | | |


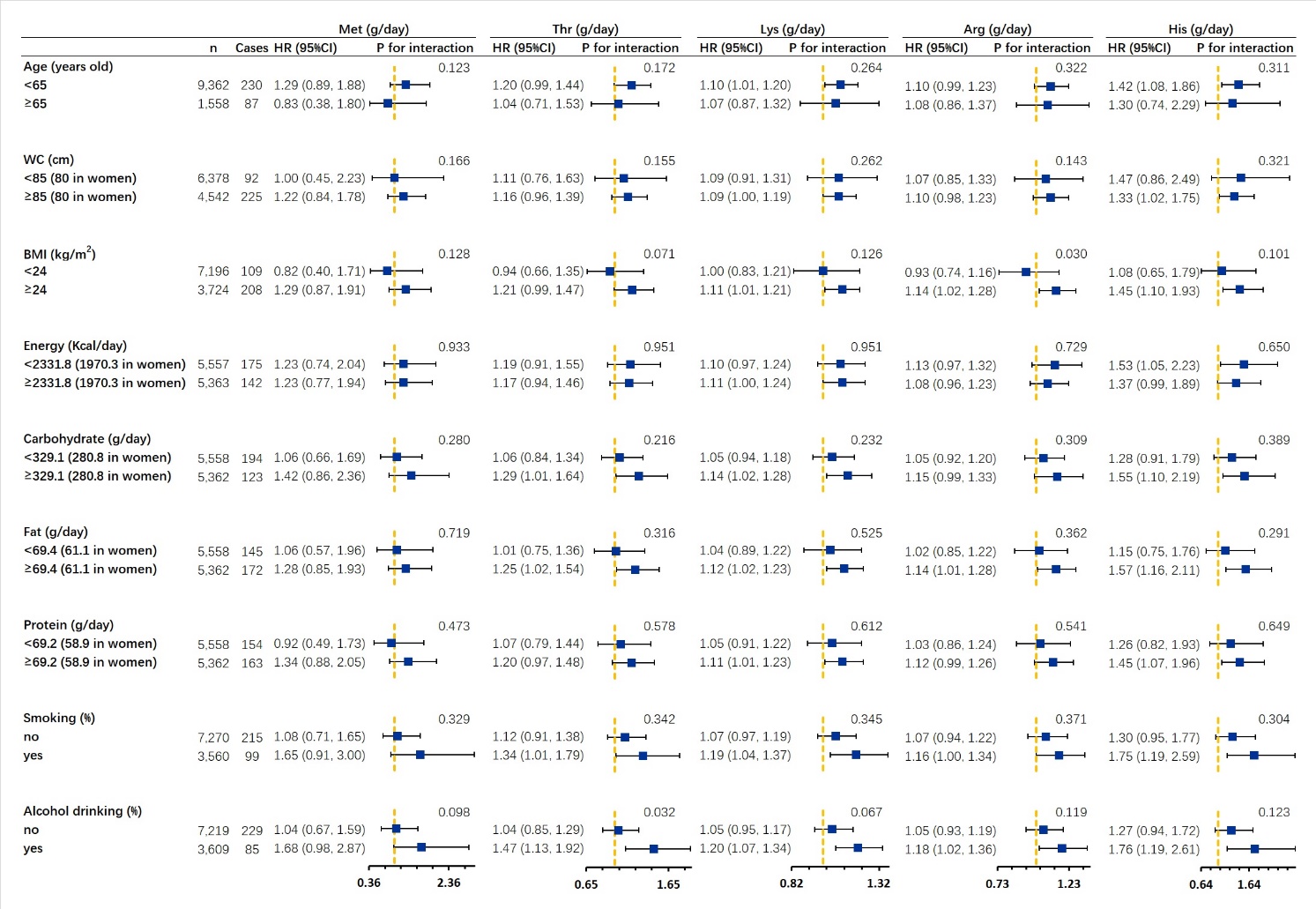
**FIGURE S1** Stratified analyses of relationship between dietary intakes of amino acids and T2DM based on confounders. Regression models were adjusted for age, nationality, education, urban residents, hypertension, physical activity, smoking, alcohol drinking, intakes of energy, carbohydrate, fat and protein. **Abbreviations:** Met: methionine; Thr: threonine; Lys: lysine; Arg: arginine; His: histidine; T2DM: type 2 diabetes mellitus; BMI: body mass index; WC: waist circumference; HR: hazard ratio; 95% CI: 95% confidence interval.
